# Characterization of Eag1 Channel Lateral Mobility in Rat Hippocampal Cultures by Single-Particle-Tracking with Quantum Dots

**DOI:** 10.1371/journal.pone.0008858

**Published:** 2010-01-25

**Authors:** David Gómez-Varela, Tobias Kohl, Manuela Schmidt, María E. Rubio, Hiroshi Kawabe, Ralf B. Nehring, Stephan Schäfer, Walter Stühmer, Luis A. Pardo

**Affiliations:** 1 Department of Molecular Biology of Neuronal Signals, Max-Planck Institute of Experimental Medicine, Göttingen, Germany; 2 European Neuroscience Institute, Göttingen, Germany; 3 Department of Molecular Neurobiology, Max-Planck Institute of Experimental Medicine, Göttingen, Germany; 4 Departments of Neuroscience and Molecular and Human Genetics, University of Texas, Houston, Texas, United States of America; 5 Institute of Biophysics/BIOTEC, Dresden, Germany; 6 Deutsche Forschungsgemeinschaft Research Center for Molecular Physiology of the Brain, Göttingen, Germany; University of Oldenburg, Germany

## Abstract

Voltage-gated ion channels are main players involved in fast synaptic events. However, only slow intracellular mechanisms have so far been described for controlling their localization as real-time visualization of endogenous voltage-gated channels at high temporal and spatial resolution has not been achieved yet. Using a specific extracellular antibody and quantum dots we reveal and characterize lateral mobility as a faster mechanism to dynamically control the number of endogenous ether-a-go-go (Eag)1 ion channels inside synapses. We visualize Eag1 entering and leaving synapses by lateral diffusion in the plasma membrane of rat hippocampal neurons. Mathematical analysis of their trajectories revealed how the motion of Eag1 gets restricted when the channels diffuse into the synapse, suggesting molecular interactions between Eag1 and synaptic components. In contrast, Eag1 channels switch to Brownian movement when they exit synapses and diffuse into extrasynaptic membranes. Furthermore, we demonstrate that the mobility of Eag1 channels is specifically regulated inside synapses by actin filaments, microtubules and electrical activity. In summary, using single-particle-tracking techniques with quantum dots nanocrystals, our study shows for the first time the lateral diffusion of an endogenous voltage-gated ion channel in neurons. The location-dependent constraints imposed by cytoskeletal elements together with the regulatory role of electrical activity strongly suggest a pivotal role for the mobility of voltage-gated ion channels in synaptic activity.

## Introduction

The dynamic molecular composition of synapses is crucial for the development and fine-tuning of nervous systems to external cues. One of the key processes of synaptic transmission is the communication along a neuron encoded in action potentials. Voltage-gated ion channels are pivotal for the generation and propagation of neuronal action potentials [1]. Importantly, it has been established that not only the biophysical features but also the spatial distribution of voltage-gated ion channels tune the signaling properties of a neuron [2].

The mechanisms of axonal and dendritic transport have been classically attributed to intracellular trafficking based on motor proteins and cytoskeletal elements [3]. However, fast changes in synaptic events are unlikely to rely on intracellular trafficking which controls the localization of synaptic receptors within a time frame of minutes [4]. In the last years, technical advances using quantum qots (QD) nanocrystals have made it possible to study the mobility of endogenous ion channels in neurons at high temporal and spatial resolution. By these means, different reports have demonstrated lateral diffusion as a mechanism to control the abundance of receptors in the postsynaptic density in the range of seconds [5]–[7] and consequently, the fidelity of synaptic transmission [4]. Despite of their physiological relevance, the existence of similar mechanisms for controlling the position of voltage-gated ion channels in the CNS is largely unknown.

In the present study, we use state of the art single-particle-tracking (SPT) techniques to demonstrate for the first time that an endogenous voltage-gated ion channel, namely Eag1 (Kv10.1), rapidly enters and exits synapses by laterally diffusing in the plasma membrane of cultured rat hippocampal neurons. Eag1 channels play an important role in synaptic physiology as suggested by the phenotype of *eag* mutants in Drosophila [8], however, their synaptic function in vertebrates is unknown. We show that Eag1 channels exhibit Brownian diffusion extrasynaptically, but get transiently trapped when they diffuse inside synapses. Furthermore, our data indicate that the mobility of Eag1 channels is highly regulated specifically inside synapses by the stability of the cytoskeleton and electrical activity.

By these means, our study demonstrates for the first time that lateral diffusion is a highly regulated mechanism that enables Eag1 channels to enter and leave synapses, and furthermore to tightly control their spatio-temporal distribution inside synaptic terminals.

## Methods

### Ethical Information

All experiments involving animals were performed with the approval of the Animal Welfare Committee of the State of Lower Saxony (Niedersächsische Tierschutzkommission). All aspects of the program for housing, management and veterinary care follow the guidelines set down in the Animal Welfare Committee of the State of Lower Saxony (Niedersächsische Tierschutzkommission).

### Hippocampal Primary Cultures

Hippocampal neuronal cultures were prepared from E18 Wistar rats and cells were plated at a density of 2×10^5^ cells/ml in Nunc chambers precoated with poly-D-lysine. Cultures were maintained in serum-free Neurobasal “A” media (Gibco) supplemented with B27 (1×; Gibco), bFGF (5 ng/ml; GibcoBRL) and L-Glutamine (500 µM; GibcoBRL). Cultures were incubated at 37°C in 10% CO_2_ for 10 DIV before being used for imaging.

### Immunostaining of Brain Slices

Rat brain saggital sections (40 µm) were cut in cold PBS using a vibratome (Leica, Vienna, Austria). Slices were blocked for 1 h with 10% normal goat serum in PBS and subsequently incubated with anti-Eag1 monoclonal antibody (mAb62; 2 µg/ml in PBS) at 4°C overnight. The protocol followed the avidin biotin-peroxidase system (Vectastain kit, Vector Laboratories, Burlingame, CA). Antibody binding was visualized with 3′-3-diaminobenzidine tetrahydrochloride (DAB; DAB substrate kit for peroxidase, Vector Laboratories). Controls were done by either omitting mAb62 or by prior incubation of mAb62 with the corresponding immunogen fusion protein (10 µg/ml final concentration) at 4°C for 24 h. Sections were analyzed with a Zeiss Axiophot microscope.

### Electron Microscopy: Postembedding Immunogold Labeling Procedure after Freeze-Substitution

mAb62 (1 µg/ml) was used at a dilution of 1∶200 and labelled with 5 nm colloidal gold-coupled secondary antibodies (Amersham, Piscataway). Controls included omitting mAb62 and preadsorption of mAb62 with the corresponding blocking protein (10 µg/ml final concentration). Electron micrographs were taken at 30.000× magnification and scanned at a resolution of 3600 dpi using a Linotype-Hell scanner (Heidelberg, Germany). Image processing was performed with Adobe Photoshop by using only the brightness and contrast commands to enhance gold particles.

### Eag1 Immunofluorescence

Hippocampal cultures were immunolabeled using antibodies against Eag1 channels (mAb62; 10 µg/ml), Tau (Santa Cruz, 1∶1000), Map2 (Santa Cruz; 1∶1000) and Synaptophysin (Sigma; 1∶1000). Surface Eag1 immunolabeling was performed after light fixation (0.1% PFA for 10 min at room temperature) by incubating the cells for 1 hour at room temperature with the indicated antibody concentrations in Ringer and 5% of Donkey serum. After several washes with Ringer, secondary antibodies coupled to FITC, Cy3 or Cy5 were added (Jackson InmunoResearch; 1∶250). The specificity of the signals was tested following the same protocol but incubating only with a secondary antibody or using a non-immunogenic IgG as primary antibody (data not shown). Afterwards, cell were fixed with 4% paraformaldehyde and permeabilized with 0.5% Triton X-100 in PBS. Primary antibodies against intracellular proteins were then added for one hour, cultures were washed and incubated with secondary antibodies conjugated with FITC, Cy3 or Cy5 dyes for two hours. Fluorescence images were acquired using a CCD camera (C4742-80-12AG, Hamamatsu Photonics Deutschland GmbH, Germany) mounted on an inverted Zeiss microscope with standard filter sets and AxioVision 4.4 software (Zeiss, Jena, Germany). The exposure time was determined to avoid pixel saturation.

For colocalization the plugin JACoP [9] of ImageJ [10] was used. For the colocalization between Synaptophysin and Eag1 three neurite segments of 20 µm were randomly selected. To avoid a biased user-defined threshold, the fluorescence signals were segmented applying a threshold value equal to the mean background fluorescence intensity ±2 S.E.M. Cell bodies were excluded for this analysis. For colocalization analysis between Eag1, Tau and Map2, we segmented the images as described above but included all labeled neurites while omitting cell bodies. For all inmunostaining experiments we analyzed 15–30 neurons from minimum 3 independent neuronal cultures.

### Lentiviral Infection of Rat Neuronal Cultures

The lentivirus expression vector encoding Munc13 in frame with a C-terminal eGFP was constructed in a modified FUGW vector with a synapsin promoter. After production of Munc13-2eGFP lentivirus, primary cultures of hippocampal neurons were prepared and infected at 4 DIV with lentivirus to express recombinant proteins.

### Live Cell Staining of Eag1 Channels

Hippocampal neuronal cultures (10 DIV) were washed twice and incubated for 20 min with PBS/0.1% BSA at room temperature. Neurons were incubated in PBS/0.1% BSA for 15 min at 37°C with 2 µg/ml of primary monoclonal antibody mAb62 for Eag1. This antibody is directed against an extracellular epitope of Eag1 channels [11]. After 3 washes with PBS/0.1% BSA, neurons were incubated for 5 min at room temperature with 25 pM F_ab_-QDs fragments (Qdot 605 goat F(ab′)_2_, Invitrogen) in PBS/0.1% BSA. Following three washes with saline buffer (160 mM NaCl, 4.5 mM KCl, 2 mM CaCl_2_, 1 mM MgCl_2_, 10 mM glucose, 10 mM HEPES pH 7,4), active presynaptic terminals were stained for 30 s with N-(3-triethylammoniumpropyl)-4-(6-(4-diethylamino)phenyl)-hexatrienyl) pyridinium dibromide (FM4-64; 4µM; Invitrogen) in saline buffer with 40 mM KCl to stimulate vesicle recycling. After three washes with saline buffer, Nunc chambers were ready for imaging sessions. Negative controls were processed identically, but were incubated only with F_ab_-QDs fragments omitting primary antibodies. All recordings were made at room temperature and taken within 20–30 min following the staining to minimize endocytosis [12]. Additionally, after this 20–30 minutes acid stripping (pH 5.5, 1min; [13]) removed >90% of Eag1-QDs complexes, indicating that we imaged events at the neuronal surface. To decrease the likelihood of tracking a Eag1-QDs complex in dendrites (around 18% in our cultures, mainly detected in cell bodies and proximal dendrites), during the recording sessions we followed exclusively Qdots moving along the surface of thin and distal neurites. For SPT experiments we used 3–6 different neuronal cultures for each experimental group.

### Microscopy and Single Particle Detection

We used an inverted microscope (Zeiss Axiovert 200M, Göttingen, Germany) equipped with a 63× oil immersion objective (NA = 1,40). Samples were illuminated with an Hg^+^ lamp and imaged with appropriate excitation filters, dichroic mirrors and emission filters, respectively: FM4-64 (BP 450–490; FT 510; BP 515–565); QD 605 (D405/20; DCLP 425; 605 WB20). Fluorescence images were acquired with 95 ms exposure time at 10 Hz using a CCD camera (C4742-80-12AG, Hamamatsu Photonics Deutschland GmbH, Germany) and AxioVision 4.4 software (Zeiss, Jena, Germany).

### Particle Tracking and Data Analysis

Tracking of fluorescent spots was performed with the ImageJ plugin ‘SpotTracker’ after processing image sequences of 900 frames with the ‘SpotEnhancing Filter’ plugin [14]. We determined diffusion coefficients for defined parts of trajectories by fitting the first 5 points of the mean square displacement (MSD) *versus* lag time t_lag_ plots [5]. For this purpose MSD curves were fitted according to Savin and Doyle [15], thereby rejecting the bias of the MSD resulting from diffusive motion during the exposure time of a single image:
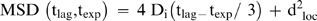
t_exp_ represents the sum of all single exposure times applied for acquisition from t = 0 to t = t_lag_ and corrects for the underestimation of D_i_ that becomes relevant if the exposure time t_exp_ is comparable to the lag time t_lag_.

SPT studies rely on sub-pixel accuracy and precision when the signal-to-noise-ratio (SNR) is around 30. However, this sub-pixel accuracy must be demonstrated with numerical simulations [16]. Therefore, our global localization accuracy (d_loc_) was determined as the sum of the accuracy of our tracking algorithm, d^2^_loc1_, and the vibrational stability of the setup, d^2^_loc2_. The latter was determined to be 8.4 * 10^−4^ µm^2^ from the MSD of immobile spots. The accuracy of our tracking algorithm was d^2^_loc1_ = 1.4 * 10^−3^ µm^2^ and was determined with a simulated movie. For simulations, parameters of the experimental setup (CCD noise and gain, t_exp_/t_lag_) as well as sample parameters (diffusion constant, spot intensity) typical for the tracking of membrane channels were employed. We determined, d^2^_loc1_ as the mean error between MSDs (step sizes 1 to 5 frames) that corresponded to the x,y spot positions after applying ‘SpotTracker’ to the simulation, and the real x,y spot positions of this simulated movie. As a result, our localization accuracy (d_loc_) was 50 nm and our resolution limit in terms of diffusion coefficients was 0.008 µm^2^/s.

To quantify the dynamic behavior of labeled channels we determined instantaneous diffusion coefficients (D_i_inst_) along trajectories. For this purpose MSD curves calculated over contiguous stretches of 10 frames were plotted and fitted. The periods where the fluorescence signal disappears due to the blinking of single quantum dots where not taking in account for our analyses. In turn, we reconstructed the whole trajectory binding the different “subtrajectories” right before and after the dark blinking periods.

In order to differentiate between synaptic and extrasynaptic trajectories, we draw regions of interest (ROI, maximun 3×3 pixels) with perimeters limited by the borders of the FM boutons. We defined synaptic and extrasynaptic trajectories for 10 or more consecutives frames where the center of the QD is inside and outside the ROI area, respectively.

The different synaptic diffusion parameters measured in this study were calculated as follows: synaptic *dwell times* were defined as the total duration of detection of Eag1-QDs channels at synapses divided by the number of exits from synapses; *time of confinement* was calculated by dividing the total time that Eag1 spent inside synapses by the total time of the recording; *number of transitions* was calculated by dividing the sum of entries and exists of synapses by the total time of the recording (in min); *diameter of confinement* was calculated by fitting the MSD of all synaptic trajectories (with 10 or more synaptic frames) to the expression for a confined diffusion:
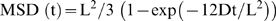
where *L* is the side of a square domain in which diffusion is supposed to be restricted. Like Charrier et al., we considered that these channels were confined in circular synaptic areas whose diameter *d*_conf_ was related to *L* by:
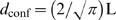



### Statistical Analysis

Statistical analysis was performed using GraphPad Prism software. Two-tailed Student's t-test was chosen only when the standard deviations (s.d.) of the compared populations were similar. The nonparametric Mann-Whitney test was used for single comparisons (if not stated otherwise) and one-way ANOVA followed by Bonferroni's test was used for multiple comparisons.

## Results

### Detection of Endogenous Eag1 Channels in Hippocampal Neurons

**Figure 1:**
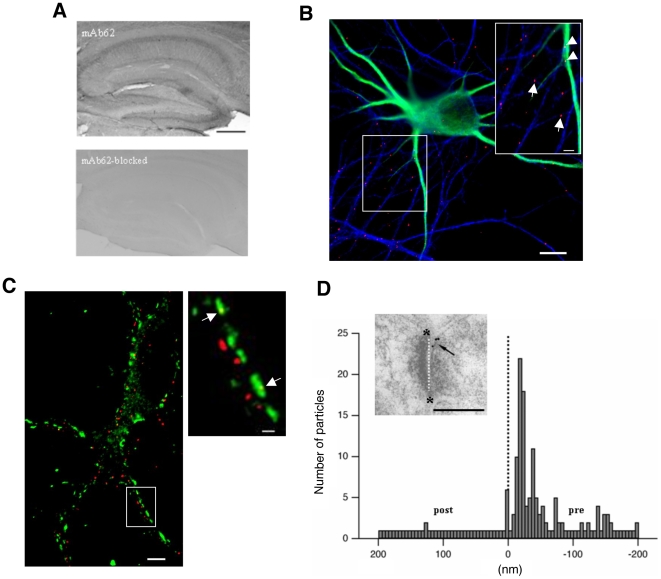
MAb62 as a tool for detecting Eag1 channels in axons of hippocampal neurons. (**A**) Immunostaining of Eag1in brain slices. MAb62 detects Eag1 in different regions of the hippocampus (mAb62). The signal disappears upon blocking mAb62 with the corresponding epitope. Scale bar: 500 µm. (**B**) Immunostaining of Eag1 (red), Tau (blue) and Map2 (green) in hippocampal neurons (scale bar 10 µm). The inset represents Eag1 clusters colocalizing with Tau (arrows) and with Map2 (arrowheads; scale bar: 3 µm). (**C**) Immunostaining of Eag1 (red) and Synaptophysin (green). The inset represents Eag1 channel clusters colocalizing with Synaptophysin (arrows; scale bar 3 µm). (**D**) The histogram depicts the axodendritic distribution of Eag1 channels in CA1 Schaffer collaterals. The analysis shows that gold particles labeling Eag1 distribute within the presynaptic terminal (n = 125 gold particles, N = 31 micrographs; black dotted line indicates the synaptic cleft position as a reference). The inset shows an electronmicrograph of a CA1 Schaffer collateral after postembedding immunogold labeling of Eag1 using mAb62. Gold particles are observed at the presynaptic membrane facing the postsynaptic membrane of dendritic spines of pyramidal cells. The white dotted line represents the cleft and asterisks represent the edges of the postsynaptic density (scale bar: 250 nm).

One of the prerequisites and bottlenecks for performing SPT studies on endogenous neuronal proteins, and in particular on voltage-gated ion channels, is the availability of antibodies that effectively and specifically label extracellular regions of these proteins [17]. In order to detect Eag1 channels in rat hippocampal neurons, we used a monoclonal antibody (mAb62) able to detect an extracellular epitope in Eag1 channels [11]. The antibody revealed the expression of Eag1 in different regions of the rat hippocampus. Of note, the signal disappeared when the antibody was blocked with its specific epitope or when omitted (Fig. 1A). Next, we characterized the expression pattern and subcellular distribution of endogenous Eag1 in our hippocampal cultures. Immunofluorescence studies demonstrated that the vast majority of Eag1 channels were primarily localized to axons (82% of colocalization with the axonal marker Tau; Fig. 1B; [18]). Only a very small fraction colocalized with the dendritic marker Map2, which applied mainly to cell bodies and proximal dendrites (Fig. 1B). Furthermore, colocalization with Synaptophysin demonstrated that 25% of axonal Eag1 was present in the membrane of presynaptic terminals (Fig. 1C and quantification in 4A). Accordingly, electron microcopy studies confirmed that Eag1 channels were present at the presynaptic membrane of CA1 Schaffer collaterals facing the postsynaptic membrane of dendritic spines of pyramidal cells (Fig 1D). No signal was detected when we blocked the antibody with the specific epitope (data not shown). In-depth characterization of the axodendritic-distribution of gold particles revealed that Eag1 predominantly localized to the presynaptic terminal including both, the plasma membrane and intracellular components (Fig. 1D).

In conclusion, our data demonstrate that mAb62 is able to detect endogenous Eag1 in rat hippocampal neurons and, therefore, serves as a tool for performing SPT studies.

### Endogenous Eag1 Channels Diffuse within the Plasma Membrane of Hippocampal Neurons

We set out to study the mobility of endogenous Eag1 channels in the plasma membrane of hippocampal neurons using SPT. To this purpose we incubated neuronal cultures at 10 days *in vitro* (DIV; [18], [19]) with low concentrations of mAb62. Primary antibody-antigen complexes were visualized with F_ab_ fragments conjugated to QD nanocrystals. By these means individual QD-Eag1 complexes were identified by their characteristic blinking and could be followed over time (Movie S1 in the Supporting Information). To assure the tracking of axonal Eag1 channels, we only recorded complexes in distal thin neurites and did not include QD-Eag1 complexes in cell bodies [20] or in proximal thick neurites. The QD signal was specific to the presence of the primary antibodies, as incubation with QD-F_ab_ fragments alone did not give any signal (data not shown).

**Figure 2:**
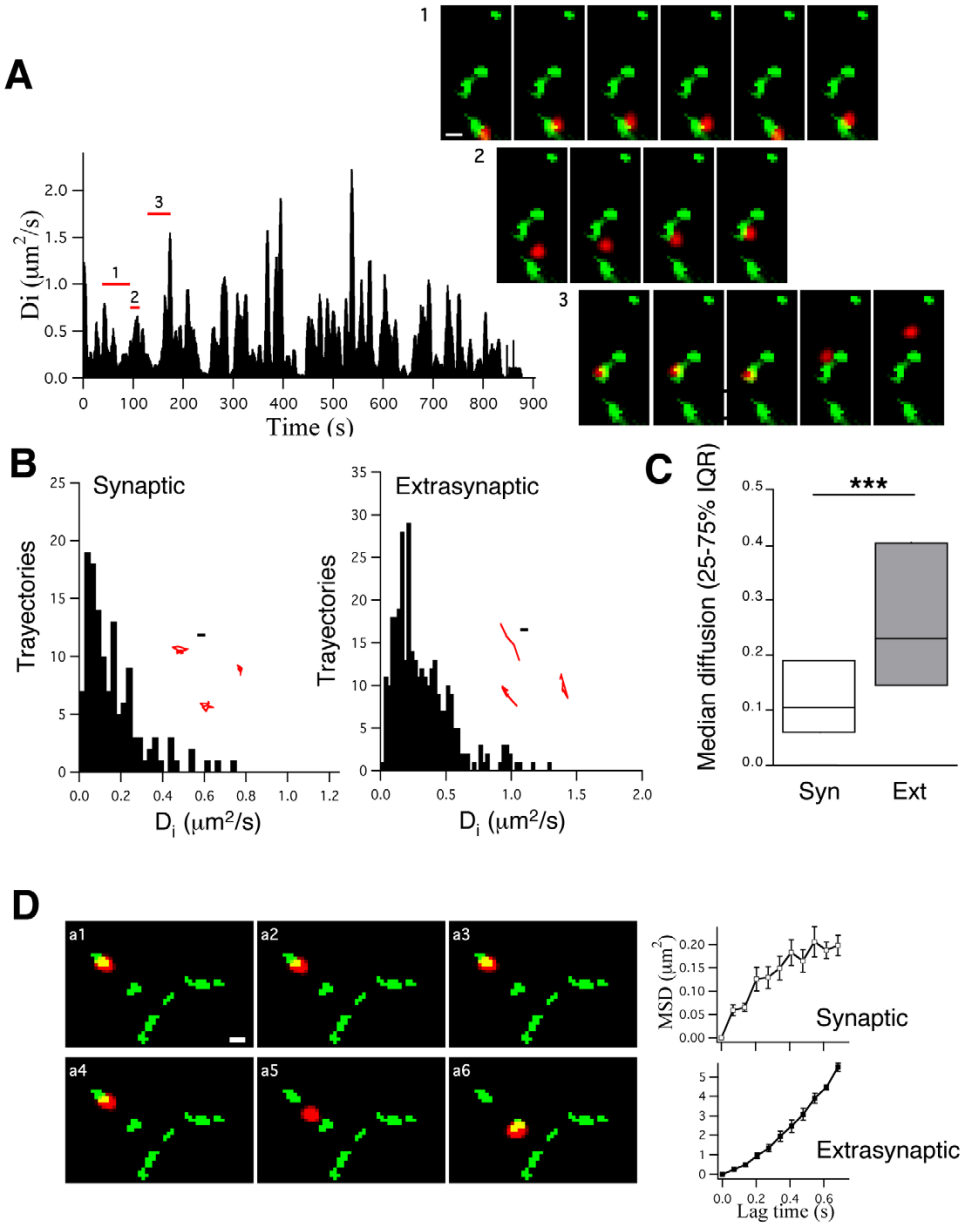
Eag1 voltage-gated potassium channels enter and leave synapses by lateral diffusion. (**A**) *Left*, histogram showing the changes of D_i_ values over time for Eag1 channels in hippocampal neuronal cultures. *Right*, consecutive frames of a representative recording showing diffusion of Eag1 channels inside a presynaptic terminal (Panel 1) and between different presynaptic terminals (Panel 2 and 3; scale bar: 1µm). (**B**) D_i_ values were fitted to MSD plots generated from either extrasynaptic or synaptic trajectories composed of at least 10 consecutive frames. After binning with a step size of 0.025 µm^2^/s the number of trajectories was plotted over D_i_ values. Insets, examples of synaptic and extrasynaptic trajectories (scale bar: 200 nm). (**C**) Median diffusion (±25–75% interquartile range) values for Eag1 channels in extrasynaptic and synaptic trajectories based on data from (B). (**D**) MSD plots *versus* time for Eag1 channels inside a synapse (upper graph, consecutive panels from a1 to a3) and outside a synapse (lower graph, consecutive panels from a4 to a6; scale bar: 1µm).

Continuous video recording at 10 Hz over 90 s revealed two different Eag1 populations based on their diffusion properties: immobile (D_i_<0.008 µm^2^/s; see Methods) or mobile (D_i_>0.008 µm^2^/s). We focused our study on the mobile population that represented the vast majority of the Eag1-QD complexes detected (70.3±0.9%, p<0.01 by Student's t-test, n = 132 QDs, 12 movies, 3 cultures; data not shown). Eag1 channels laterally diffuse within the plasma membrane (Fig. 2A; Movie S1) and thereby alternate their position between extrasynaptic and synaptic regions. We tracked the movement of QD-Eag1 complexes that crossed a synaptic/extrasynaptic border at least twice during their trajectory. Synaptic terminals were identified as FM 4-64 positive boutons (green boutons in Movie S1 and in Panels of Fig. 2A; [5]). Eag1 channels either moved between different presynaptic terminals or in a confined region inside a synaptic bouton (Panels in Fig. 2A). The signal-to-noise ratio of our recordings (Figure S1 in the Supporting Information) and various error sources for the calculation of spot positions (see Material and Methods) resulted in a point accuracy of 50 nm and a resolution limit of 0.008 µm^2^/s for diffusion coefficients (D_i_). Quantification of the movements of Eag1 channels showed that their diffusion coefficients changed between 0.008 and 2 µm^2^/s (Fig. 2A and 2B), which agrees with the values reported for different ligand-gated ion channels [5], [6], [21], [22] and tagged overexpressed Kv ion channels [23]. In-depth characterization revealed that Eag1 channels diffused slower inside (median D_i_ = 0.104 µm^2^/s, interquartile range (IQR) = 0.057–0.189 µm^2^/s, n = 155) than outside presynaptic terminals (median D_i_ = 0.23 µm^2^/s, IQR = 0.14–0.40 µm^2^/s, n = 453 trajectories from 3–5 independent cultures; Mann-Whitney test, *** p<0.001; Fig 2B and 2C). Interestingly, the trajectories of Eag1 were more confined inside presynaptic terminals (insets in Fig 2B), suggesting restricted movement in synapses. In order to test this hypothesis, we analyzed the shape of the MSD plots for both, synaptic and extrasynaptic trajectories (Fig 2D; [24]). As defined by Kusumi and collaborators [24], the asymptotic shape demonstrated that Eag1 channels exhibited restricted diffusion inside presynaptic terminals, while the linear shape indicated Brownian diffusion in extrasynaptic trajectories. Figure 2D represents a reversible diffusion process: an Eag1 channel shows restricted movement when localized at synapses (upper-right graph in Fig. 2D corresponding to consecutive Panels from a1 to a3) but switches to free Brownian diffusion upon lateral movement out of the synaptic bouton (lower-right graph in Fig. 2D corresponding to consecutive Panels from a4 to a6).

**Figure 3:**
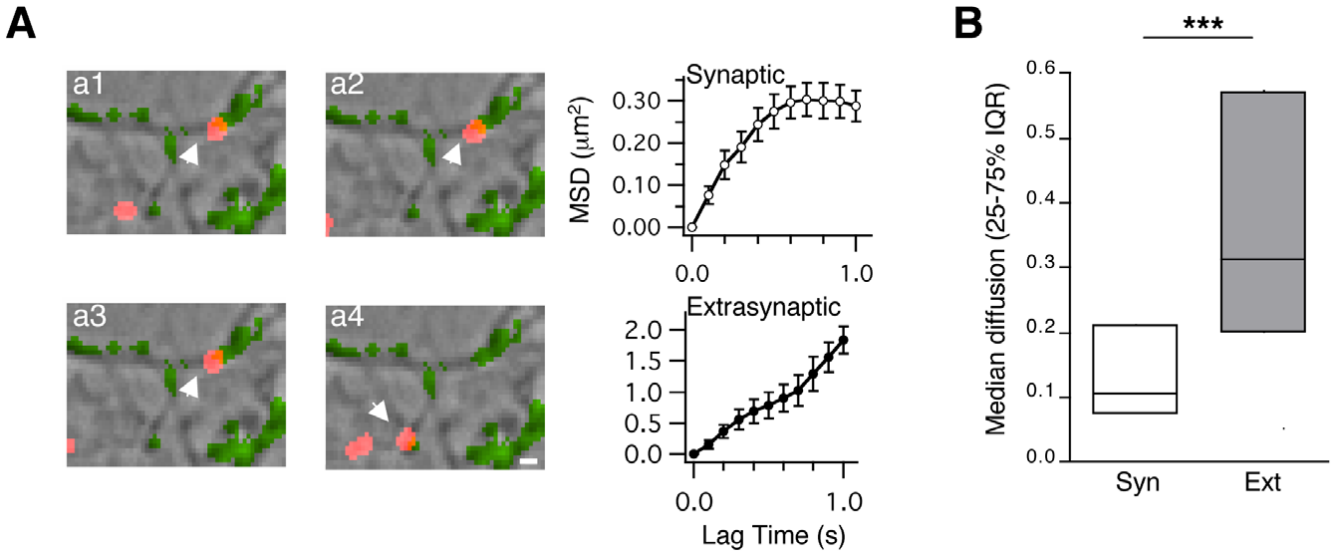
Eag1 channels laterally diffuse in axons labeled with Munc-13eGFP. (**A**) Overlay images (brightfield, Munc-13eGFP in green and QD-Eag1 in red) of a representative QD-Eag1 recording (arrows). Right graphs, MSD plots *versus* time for Eag1 channels inside (upper, consecutive panels from a1 to a2) and outside a synapse (lower, consecutive panels from a3 to a4; scale bar: 1 µm). (**B**) Median diffusion plots (±25–75% interquartile range) for Eag1 channels in synaptic and extrasynaptic trajectories.

The axonal nature of the Eag1 channels tracked in this study was confirmed by studying the lateral diffusion of Eag1 channels in neurons transfected with a well-established presynaptic marker, Munc-13-eGFP [25]. After retroviral infection with Munc-13-eGFP (see Material and Methods) we again observed that Eag1 channels laterally diffused in the axonal membrane of hippocampal neurons (Fig. 3A and Movie S2 in the Supporting Information). Importantly, we measured similar diffusion characteristics for the mobility of Eag1 channels when Munc-13-eGFP was used as a presynaptic marker instead of FM 4-64 (median = 0.10 µm^2^/s, IQR = 0.07–0.2 µm^2^/s, n = 98 and median = 0.31 µm^2^/s, IQR = 0.19–0.57 µm^2^/s, n = 121 for synaptic and extrasynaptic trajectories from 3–5 independent cultures, respectively; Mann-Whitney test, *** p<0.001; Fig. 3A and 3B).

In summary, our data demonstrate that endogenous presynaptic Eag1 channels, enter and leave synapses by lateral diffusion. These channels diffuse freely outside presynaptic terminals but get transiently tethered inside synapses. This last finding is highly indicative of fine molecular mechanisms with the potential to quickly control the dynamic localization of voltage-gated ion channels inside synapses.

### F-Actin Disruption, Microtubule Depolymerization and Electrical Network Activity Regulate the Diffusion of Eag1 Channels

In order to shed light on the identity of these molecular constraints, we investigated the role of the cytoskeletal network. Disruption of actin filaments and microtubule stability has been described to regulate the number of glycine receptors inside synapses by changing their diffusion properties [12]. Furthermore, cytoskeletal elements strongly influence the biophysical properties of Eag1 channels [26].

**Figure 4:**
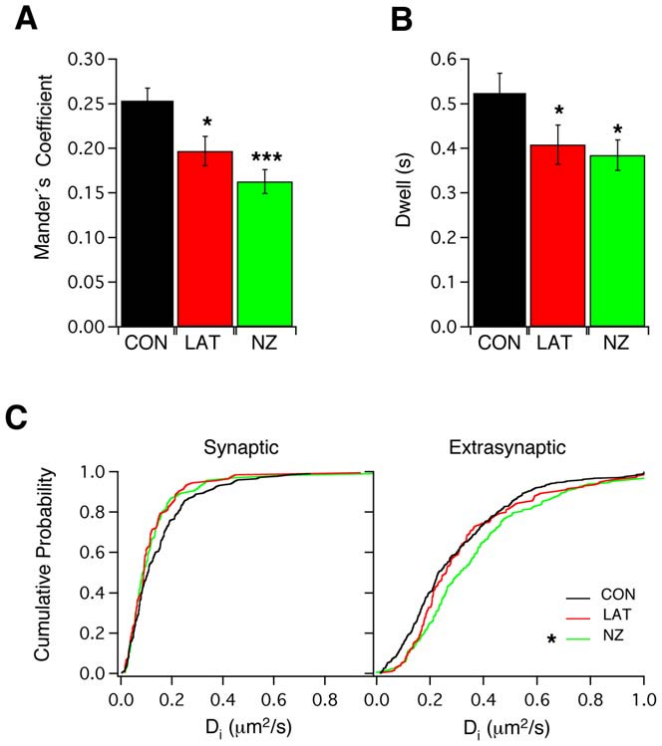
Disruption of actin filaments and microtubule stability have a profound effect on the mobility if Eag1 inside synapses. (**A**) Effect of Latrunculin A and Nocodazole on the degree of colocalization between Eag1 and Synaptophysin. (**C**) Effects of Latrunculin A and Nocodazole on synaptic dwell time. (**D**) Cumulative probabilities of D_i_ for synaptic and extrasynaptic Eag1 trajectories upon Latrunculin A and Nocodazole treatments.

We found that disruption of F-actin (by Latrunculin A, 3 µM, 1 hour; [12]) or microtubules (by Nocodazole, 10 µM, 1 hour; [12]) reduced the number of Eag1 channels inside synaptic terminals as assessed by colocalization of Eag1 with the presynaptic marker synaptophysin (Mander coefficients = 0.25±0.01, n = 19, 0.19±0.01, n = 18 and 0.16±0.01, n = 16 for DMSO, Latrunculin A and Nocodazole, respectively; one-way ANOVA followed by Bonferroni's multiple comparison test, * p<0.05, *** p<0.001; Fig 4A). In accordance with these results, our SPT studies revealed that both treatments significantly reduced the synaptic dwell time of Eag1 (0.52±0.04 s, n = 23, 0.39±0.04 s, n = 11 and 0.37±0.04 s, n = 12, for DMSO, Latrunculin A and Nocodazole, respectively; one-way ANOVA followed by Bonferroni's multiple comparison test, * p<0.05, Fig 4B). Interestingly, although the time of confinement was not changed (14.2±2.1 s, n = 23, 18.2±2.8 s, n = 11 and 14.9±2.3 s, n = 12, for DMSO, Latrunculin A and Nocodazole, respectively; one-way ANOVA followed by Bonferroni's multiple comparison test, Figure S2A), cytoskeleton disruption increased the number of transitions between synaptic and extrasynaptic spaces (33.2±5.3, n = 23, 59±8.5, n = 11 and 55±10.3, n = 12, for DMSO, Latrunculin A and Nocodazole, respectively; one-way ANOVA followed by Bonferroni's multiple comparison test, * p<0.05, Figure S2B).

Interestingly, the diffusion coefficient of Eag1 was not affected by disruption of F-actin. In contrast, depolymerization of microtubules after Nocodazole treatment increased the diffusion of Eag1 channels outside synapses (median = 0.23 µm^2^/s, IQR = 0.14–0.4 µm^2^/s, n = 453 and median = 0.282 µm^2^/s, IQR = 0.12–0.36 µm^2^/s, n = 100 for Control and Nocodazole, respectively; Mann-Whitney test, * p<0.05; Fig. 4C). In summary, our data indicate that the cytoskeletal network can regulate the diffusion of Eag1 channels and their number inside synapses most probably by controlling the time the channels interact and are restricted with/by unknown synaptic components once they enter synapses.

**Figure 5:**
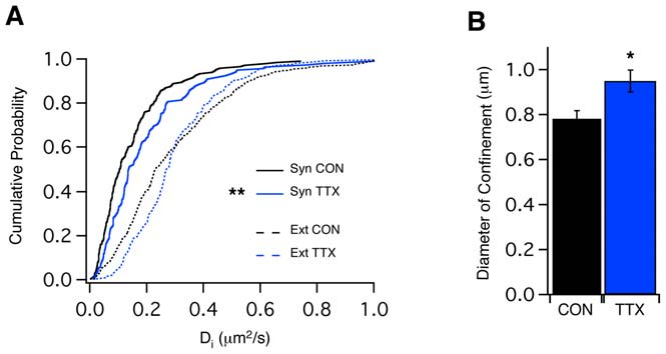
TTX acelerates Eag1 mobility inside synapses. (**A**) Cumulative probabilities of D_i_ for synaptic (full lines) and extrasynaptic (dotted lines) Eag1 trajectories upon TTX (blue) or Control (black) conditions. (**B**) Histogram represents the diameter of confinement calculated as explained in Methods.

Eag1 potassium channels activate near the neuronal resting membrane potential and therefore are important in regulating neuronal excitability [8], [27]. Furthermore, Eag channels exhibit a unique biophysical feature among all known ion channels referred to as the Cole-Moore effect. Thus, the rate of Eag channel activation strongly depends on the resting potential: hyperpolarization slows the activation kinetics down while depolarization accelerates it [28]. Therefore, we aimed to study whether electrical network activity could influence the mobility of Eag1 channels. Interestingly, we found that shutting down electrical activity by application of Tetrodotoxin (TTX, 1 µM for 24 hours; [7]) specifically accelerated the diffusion of Eag1 inside presynaptic terminals (median D_i_ = 0.104 µm^2^/s, IQR = 0.057–0.189 µm^2^/s, n = 155 and median D_i_ = 0.14 µm^2^/s, IQR = 0.08–0.26 µm^2^/s, n = 123 for Control and TTX, respectively; Mann-Whitney test, ** p<0.01; Fig 5A, solid lines), while the extrasynaptic mobility was not altered (Fig 5A, dotted lines). Accordingly, we observed that Eag1 channels are less confined and can explore a bigger synaptic area upon TTX treatment (mean diameter = 0.78±0.03 µm, n = 133 and mean diameter = 0.92±0.04 µm, n = 82 for Control and TTX, respectively; Student's t-test * p<0.05; Fig 5B).

## Discussion

The characterization of protein dynamics plays a crucial role to understand physiological processes on a molecular scale. In this study, we demonstrated for the first time how an endogenous voltage-gated ion channel, namely Eag1 (Kv10.1), enters and leaves synapses laterally diffusing within the membrane of hippocampal neurons. This finding is of high relevance as in the last years the notion has evolved that both, the number and localization of voltage-gated ion channels influence fast processes like neuronal electrical activity. However, synaptic access of endogenous voltage-gated ion channels has so far merely been attributed to relatively slow intracellular transport mechanisms [2]. Therefore, our findings suggest lateral diffusion as a means by which voltage-gated ion channels might quickly influence synaptic transmission [4].

We show that the mobility of Eag1 is slower inside than outside synapses, and that synaptic trajectories are more confined than extrasynaptic ones. Importantly, they exhibited restricted diffusion inside but Brownian diffusion outside presynaptic terminals. Of note, although widely used in the SPT field, our labeling strategy (antibody plus a F_ab_-QD fragments) might affect the accuracy of the synaptic Di values calculated [29]. Overall, lateral diffusion seems to be spatially regulated in the presynaptic terminal similar to the previously described diffusion of postsynaptic receptors [30].

Eag1 did not only slow-down, but got also transiently stabilized inside synapses. Considering that the distribution of voltage-gated ion channels is crucial for synaptic activity [2], it becomes very important to reveal potential interactions with presynaptic components that might control the diffusion of Eag1 channels inside synapses. Interestingly, the cytoskeleton has been reported to be involved in controlling the lateral diffusion of postsynaptic receptors [12], [31]. Furthermore, cortical actin seems to be an important player in anchoring transmembrane molecules by compartmentalizing the cell surface in microdomains [30], [32]. Our data show that the actin cytoskeleton and microtubule stability have a profound effect on both, the lateral mobility of Eag1 channels inside synapses and the number of synaptic Eag1 channels. This result is of high interest as it strongly suggests that changes in the mobility of Eag1 might be of physiological relevance for the role of Eag1 channels in neuronal excitability.

The underlying mechanisms of the observed changes in lateral diffusion could be as manifold as the role of the actin cytoskeleton in synapse organization itself. The presynaptic terminal harbors the cytomatrix at the active zone (CAZ; [33], [34]), which exhibits a complex architecture of cytoskeletal and membrane-bound scaffolding proteins believed to be responsible for the organization of the machineries for membrane trafficking processes [35], [36]. This actin network underneath the presynaptic plasma membrane might act as a barrier for the access of vesicles [37], [38] and proteins to the active zone membrane [39]. Following this model, disruption of the cytoskeleton might reduce the number of physical barriers inside the presynaptic terminal thereby reducing the physical constraints for Eag1 channels to diffuse in and out synapses. Accordingly, we demonstrate that cytoskeletal disruption reduced the dwell time of Eag1 channels and the total number of Eag1 channels inside synapses.

Camacho and colleagues reported increased activity of Eag1 channels after disruption of the cytoskeleton [26]. Interestingly, disassembly of actin filaments increases both, the activity and the number of BK channels in presynaptic terminals of hippocampal neurons [40]. In respect to these results, our data open the exciting possibility that the cytoskeleton might regulate the diffusion of the Eag1 channels not only via direct physical interactions (“brake model”; [30]), but also by regulating the activity status of the channel. In turn this could be translated in conformational changes of Eag1 shaping the interaction with other yet to be discovered presynaptic proteins and hence its mobility. Finally, we can not exclude the possibility that cytoskeletal elements might play a role in regulating the number of Eag1 channels and/or altering the subunit composition of the complexes that are labeled with a single QD in our study [30], Neuronal electrical properties depend on the activation of ion channels at the appropriate location within the neuron [2]. As indicated before, Eag channels exhibit the unique biophysical feature named Cole-Moore effect. Therefore, this unique quality might slow down or accelerate action potential repolarization and thereby modulate firing frequencies. In fact, *eag* mutants of *Drosophila* show spontaneous repetitive firing and increased transmitter release [8]. This pronounced hyperexcitability indicates a major role of Eag channels for maintaining baseline neuronal excitability. In respect to Eag1 physiology this model would predict that among the total population of neuronal Eag1 channels, especially the dynamics of the synaptic pool must be tightly regulated. Interestingly, the majority of the regulatory effects described in this study exclusively affect the mobility of Eag1 inside synapses, which strongly indicates a sophisticated regulation for the dynamics of Eag1 channels once these channels enter synaptic terminals.

Taken together, our study demonstrates that lateral diffusion might serve as a mechanism to quickly adjust the access of Eag1 ion channels to synaptic terminals and might therefore play a pivotal role for synaptic function and plasticity. Recently the notion has evolved that receptor trafficking might underlie neurological diseases [41]. In this context, our results represent a first step toward an in-depth characterization of the mobility of voltage-gated ion channels. Future investigations of the molecular network controlling the mobility of these ion channels in neurons might reveal whether pathological changes in channel mobility contribute to channelopathies.

## Supporting Information

Figure S1 3D surface plot of a representative QD-Eag1 complex tracked in our experiments. The left top panel shows the same fluorescence signal in 2D dimension. Both images represent raw data in order to show the high signal-to-noise ratio of our recordings.
(1.56 MB TIF)

Figure S2 Effect of Latrunculin A and Nocodazole on the time of confinement (A) and the number of transitions (B) for Eag1 channels.
(0.51 MB TIF)

Movie S1 Lateral diffusion of QD-Eag1 channels (red) on the surface of a hippocampal neurons (900 continuous images at 10 frames per second). Green boutons represent presynaptic terminals labeled with FM4-64.
(1.23 MB MOV)

Movie S2 Lateral diffusion of QD-Eag1 channels (red) at Munc-13-eGFP boutons (green) on the surface of hippocampal neurons (DIC image).
(3.98 MB MOV)
