# Supplementary figures and images for: Characterization of Eag1 Channel Lateral Mobility in Rat Hippocampal Cultures by Single-Particle-Tracking with Quantum Dots
Source: PLoS One. 2010 Jan 25;5(1):e8858. doi: 10.1371/journal.pone.0008858 (PMC2810327; doi:10.1371/journal.pone.0008858)

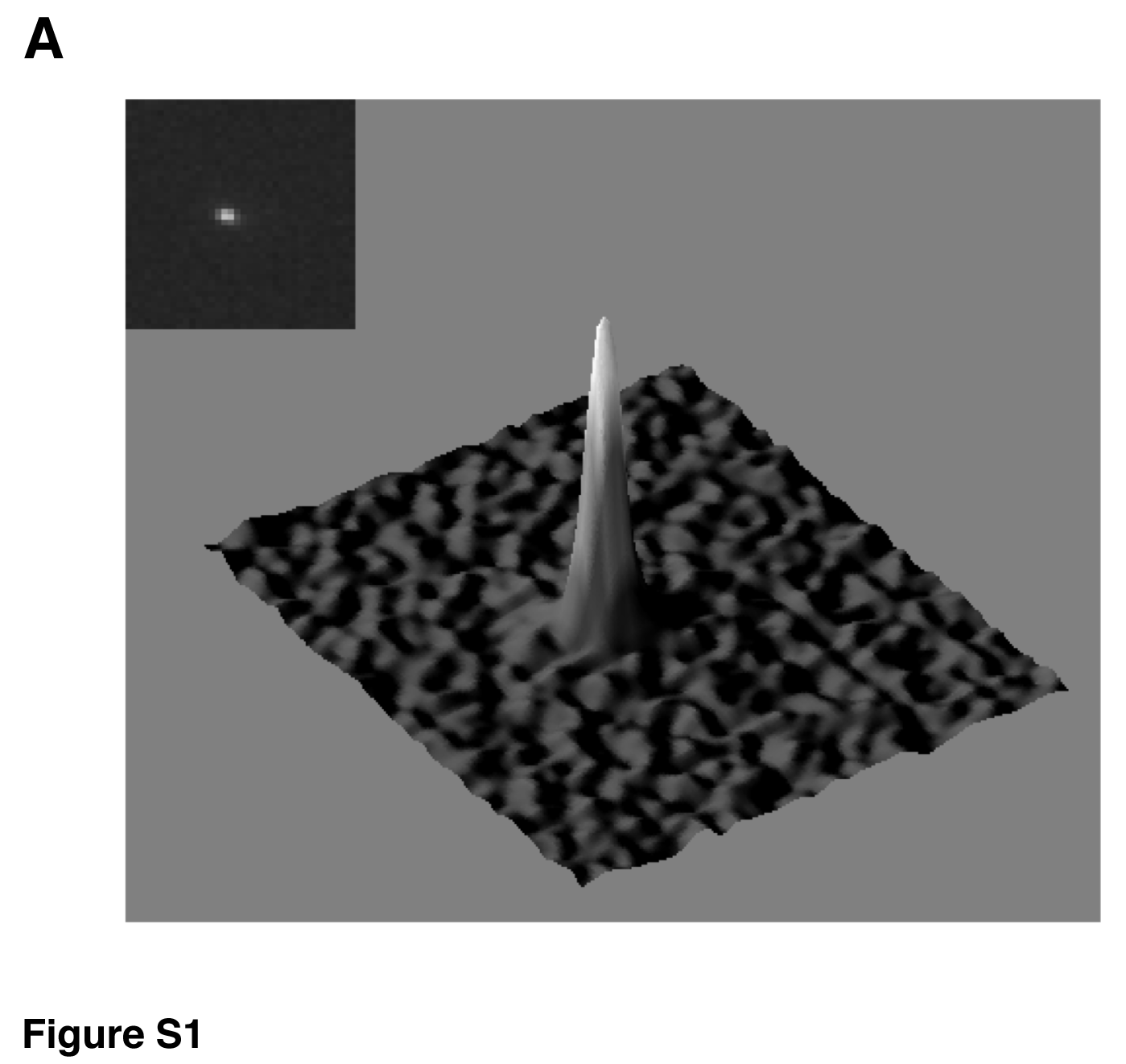

Supplement: Figure S1 — 3D surface plot of a representative QD-Eag1 complex tracked in our experiments. The left top panel shows the same fluorescence signal in 2D dimension. Both images represent raw data in order to show the high signal-to-noise ratio of our recordings. (1.56 MB TIF) [file pone.0008858.s001.tif]

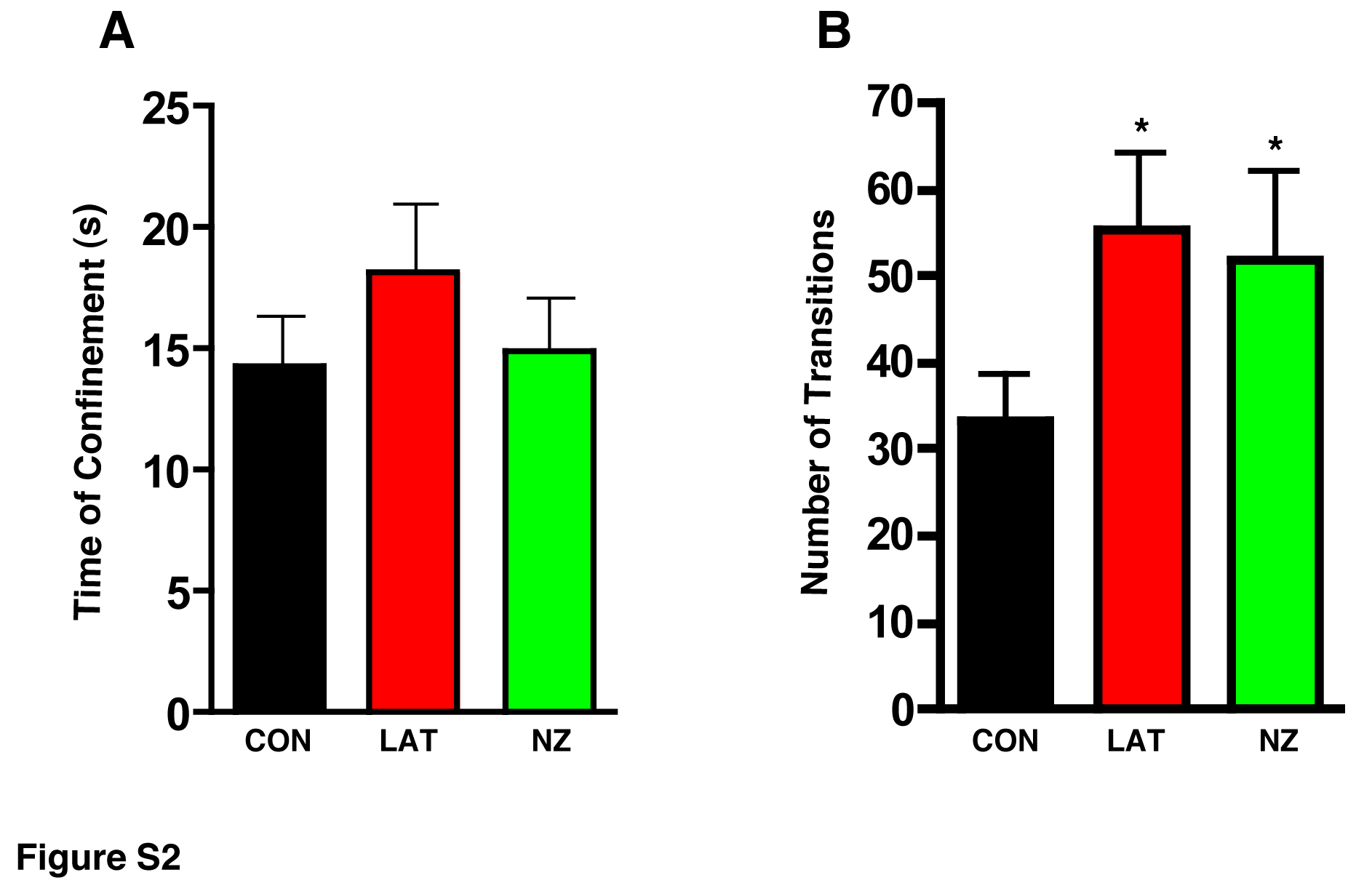

Supplement: Figure S2 — Effect of Latrunculin A and Nocodazole on the time of confinement (A) and the number of transitions (B) for Eag1 channels. (0.51 MB TIF) [file pone.0008858.s002.tif]
